# Increased Physical Activity Not Decreased Energy Intake Is Associated with Inpatient Medical Treatment for Anorexia Nervosa in Adolescent Females

**DOI:** 10.1371/journal.pone.0061559

**Published:** 2013-04-18

**Authors:** Janine Higgins, Jennifer Hagman, Zhaoxing Pan, Paul MacLean

**Affiliations:** 1 School of Medicine, Department of Pediatrics, University of Colorado, Anschutz Medical Campus, Aurora, Colorado, United States of America; 2 School of Medicine, Department of Psychiatry, University of Colorado, Anschutz Medical Campus, Aurora, Colorado, United States of America; 3 Research Institute, Children’s Hospital Colorado, Aurora, Colorado, United States of America; 4 School of Medicine, Division of Endocrinology, Diabetes and Metabolism, University of Colorado, Anschutz Medical Campus, Aurora, Colorado, United States of America; Pennington Biomedical Research Center/LSU, United States of America

## Abstract

There is a dearth of data regarding changes in dietary intake and physical activity over time that lead to inpatient medical treatment for anorexia nervosa (AN). Without such data, more effective nutritional therapies for patients cannot be devised. This study was undertaken to describe changes in diet and physical activity that precede inpatient medical hospitalization for AN in female adolescents. This data can be used to understand factors contributing to medical instability in AN, and may advance rodent models of AN to investigate novel weight restoration strategies. It was hypothesized that hospitalization for AN would be associated with progressive energy restriction and increased physical activity over time. 20 females, 11–19 years (14.3±1.8 years), with restricting type AN, completed retrospective, self-report questionnaires to assess dietary intake and physical activity over the 6 month period prior to inpatient admission (food frequency questionnaire, Pediatric physical activity recall) and 1 week prior (24 hour food recall, modifiable activity questionnaire). Physical activity increased acutely prior to inpatient admission without any change in energy or macronutrient intake. However, there were significant changes in reported micronutrient intake causing inadequate intake of Vitamin A, Vitamin D, and pantothenic acid at 1 week versus high, potentially harmful, intake of Vitamin A over 6 months prior to admission. Subject report of significantly increased physical activity, not decreased energy intake, were associated with medical hospitalization for AN. Physical activity and Vitamin A and D intake should be carefully monitored following initial AN diagnosis, as markers of disease progression as to potentially minimize the risk of medical instability.

## Introduction

Anorexia Nervosa (AN) is a complex psychiatric disorder, associated with significant weight loss accompanied by body image distortion, fear of weight gain, extreme preoccupation with losing weight, and loss of menses in females [1], [2]. AN has the highest premature mortality rate of any psychiatric disorder [3] and is increasing in prevalence amongst children and adolescents around the globe [4]. AN treatment is more successful for younger patients with shorter duration of disease and higher BMI than older patients with longer disease duration [5], [6]. Therefore, early diagnosis and effective interventions leading to successful weight restoration are crucial to stem the prevalence of this disease and attenuate the severity and recidivism in those affected. Currently, there is no dietary weight restoration protocol that serves as a standard treatment for AN. Although all AN treatments utilize hypercaloric diets, the composition, energy density, and approaches to refeeding differ markedly between hospitals and treatment facilities [7].

It is suspected that progressive dietary depletion, combined with increased activity levels, leads to physiological changes which become central to the onset and maintenance of AN. Compared to controls, women with AN consume less calories [8]–[11] with some studies showing that AN patients restrict fat intake and have higher carbohydrate and protein intakes [9], [10], [12]–[14] whereas others show equal restriction of all macronutrients such that the protein:fat:carbohydrate ratio of the diet is unchanged but total energy intake is lower relative to controls [8], [10], [11]. The only prospective study to examine diet prior to inpatient treatment conducted statistical analysis only on AN versus controls prior to diagnosis and again at diagnosis [10]. No prospective data analysis was done on AN cases over time so this study cannot describe the changes in diet that lead to inpatient treatment for AN.

This study was conducted to describe the changes in diet and physical activity that precede inpatient medical treatment for AN in adolescent females. This information can be used to inform clinical care and to investigate optimal weight restoration strategies for the treatment of AN in this population. It was hypothesized that inpatient medical treatment for AN would be associated with: 1) extreme energy restriction with selective exclusion of fat and carbohydrate in the diet; and 2) increased total amounts of physical activity.

## Materials and Methods

### Participants and Recruitment

This study was conducted according to Declaration of Helsinki guidelines and all procedures were approved by the University of Colorado Denver Combined Institutional Review Board (COMIRB). All participants provided written assent with the written informed consent of a parent or guardian.

Primary inclusion criteria were: a) females between 10 and 21 years of age, b) first admission to the Children’s Hospital Colorado Eating Disorders Program for inpatient medical care for a primary diagnosis of AN, and c) the ability to read and understand English at a 4^th^ grade level. Subjects were recruited within 5 days of medical admission. Control subjects were not recruited as this was designed to examine the dietary and physical activity patterns that lead to medical admission only in AN patients. Data from AN subjects was compared to both dietary recommended intake (DRI) for females 14–18 years [15] and average US dietary intake for females 12–19 years [16].

All patients who met the study inclusion criteria were approached for consent/assent. A total of 25 patients were approached and 20 agreed to participate. Of the five patients who did not participate, four stated that they were not interested in completing the questionnaires, the fifth had a parent who refused to sign the consent form.

### Questionnaires

This was a retrospective study which utilized self-report questionnaires to collect data. Four questionnaires were issued under the supervision of a Pediatric Clinical Translational Research Center (CTRC) Nutritionist: 1) Rockett Youth and Adolescent food frequency questionnaire (FFQ [17]), 2) 24 h food recall [18], 3) Pediatric physical activity recall (PDPAR [19]), and 4) modifiable activity questionnaire (MAQ [20]). Due to the nature of the instruments used to measure dietary intake and physical activity in this study, it is not possible to measure progressive changes over time as these questionnaires can only collect data cumulatively over 6 months (FFQ and PDPAR) and then acutely one week prior to admission (24 h food recall and MAQ). To encourage accurate reporting by subjects, those administering questionnaires emphasized during consent and prior to questionnaires that they were not part of the subject’s clinical care team, that data obtained would not be used to inform clinical care decisions, and that the clinical care team would not see data that could be traced to an individual.

The FFQ and PDPAR assessed habitual food intake and physical activity over a six month period. The 24 h food recall and MAQ assessed food intake and physical activity acutely, one week prior to admission for AN treatment. The 24 h food recall and MAQ were based on the same day during the week prior to admission. Although this is not the standard way to administer these questionnaires, data attained from the traditional method of gathering information from the 24 h period immediately prior to questionnaire administration was not relevant as subjects had already been admitted to the hospital for treatment in the previous days. Therefore, data collected in the traditional manner would have been reflective only of the hospital refeeding diet and limited physical activity, typically bed rest. Subjects had no hesitation in picking a day from the previous week as most of them stated that their food intake had been exactly the same every day. Under these circumstances, we asked them to choose a normal weekday with typical work/school patterns and exercise routines. 24 h food recalls were conducted in a forward time order (breakfast through dinner), open meal format by a trained pediatric CTRC nutritionist [18].

The PDPAR assesses only physical activity whereas the MAQ assigns METS to both physical activity (eg. riding a bike, jogging, dancing, etc) and sedentary activities (watching TV, reading, eating, etc). In order to make the scores from the MAQ and the PDPAR directly comparable, no sedentary activities were assigned METS during MAQ scoring.

### Statistical Analysis

Summary statistics were calculated for dietary intake and physical activity. The difference between parameters at the two time intervals were assessed using paired t-tests for continuous variables. Adjustment for multiple comparisons was achieved by applying the Bonfferoni adjustment to the analyses for micronutrients. All data are presented as mean ± SEM, except subject characteristics which are shown as mean ± SD.

## Results

**Figure 1:**
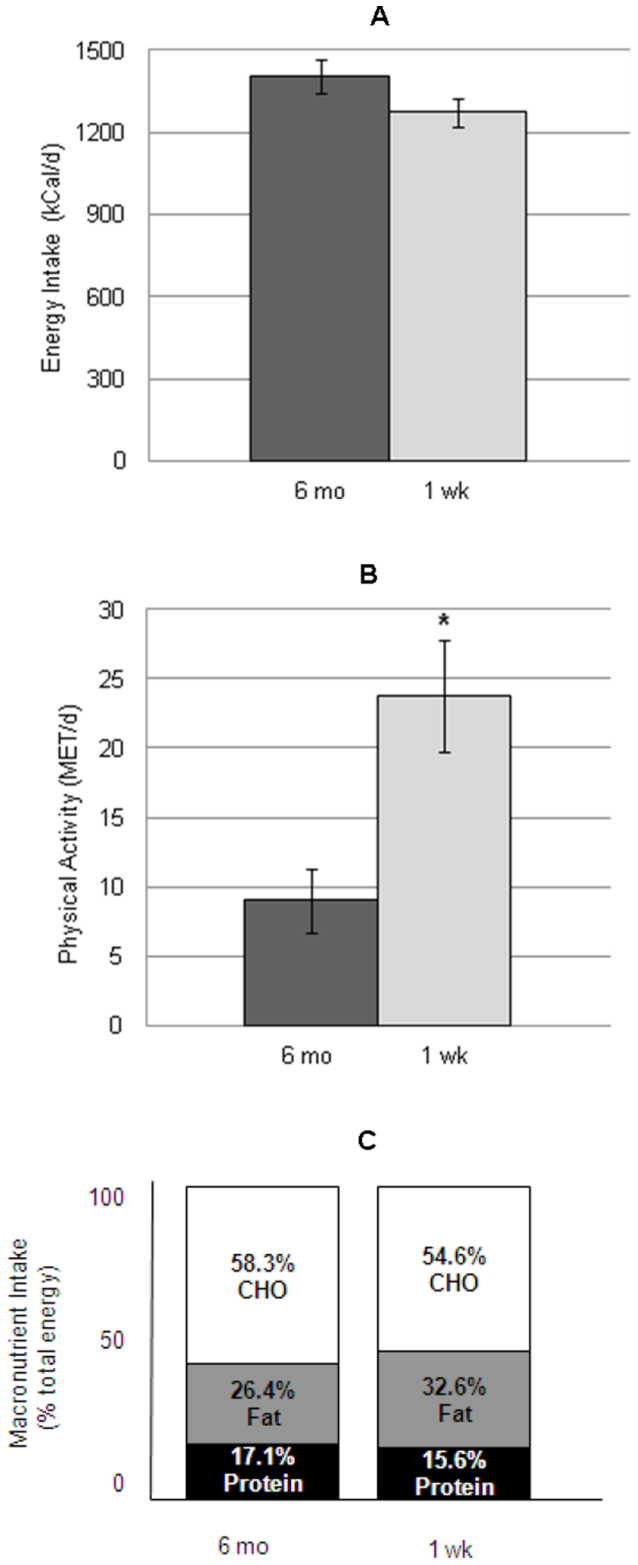
Dietary Intake and Physical Activity in Female Adolescents with Anorexia Nervosa Who Require Inpatient Medical Treatment. Total daily energy intake (A), physical activity (B), and macronutrient intake (C) over 6 months and 1 week prior to inpatient medical treatment.

Twenty females, 11–19 years of age (mean 14.3 yrs ± SD 1.8), with restricting type AN were enrolled in and completed the study. Subject characteristics are described in Table 1. There was no significant difference in reported energy intake over 6 months and 1 week prior to admission (1404±157 vs 1272±237 kcal, p = 0.312; Figure 1). Also, the macronutrient content of the diet did not change over time (Figure 1). However, physical activity increased significantly from 9.03±2.34 MET/d over 6 months prior to admission to 23.78±3.99 MET/d one week prior to admission (p = 0.0012; Figure 1).

**Table 1:** Subject Characteristics.

Age (yr)	14.3±1.8
Height (cm)	1.6±0.0
Weight at admission (kg)	38.0±1.7
Documented Duration of Illness (mo)	12.1±3.2 Median = 6±14
Admission %IBW	75.5±0.0
Admission BMI	14.9±0.4
Admission BMI z-score	−2.7±0.3
Calculated DCN at admission (kcal)	1257.2±23.6

IBW, ideal body weight for height based on Centers for Disease Control (CDC) growth charts for girls aged 2–20 years; DCN, daily caloric needs for weight stability calculated from height and weight using the Schofield equation.

Qualitatively, all subjects (100%) engaged in aerobic activity both over 6 mo and 1 wk prior to admission whereas only four subjects (20%) engaged in resistance training (weight or circuit training). The most common aerobic activities were running, bicycling, dance, swimming, and volleyball. Over 6 mo, most subjects were involved in at least three different types of activity, about 75% of which were performed at moderate or high intensity. At one week, subjects had added many activities and generally participated in four to 12 activities, about 40% of which were performed at moderate or high intensity, implying that the physical activity that was added over time was of lower intensity.

Although there was no change in reported total energy or macronutrient intake between 6 months and 1 week prior to admission, significant changes in reported micronutrient intake occurred (Table 2). Many of these changes were statistically significant, including copper, zinc, iron, and most B group vitamins (Table 2) but possibly without biological relevance as intake remained within normal limits based on Dietary Recommended Intake (DRI) for 14–18 year old females [15]. However, a few micronutrients that showed change may have biological importance. These include: Vitamin A, Vitamin D, and pantothenic acid. Total dietary calcium and phosphorus intakes did not change between 6 mo and 1 week (798±69 and 748±122 mg/d for calcium, respectively; 994±98 and 874±123 mg/d for phosphorus, respectively). Values for these nutrients closely match average US consumption which is 878±40.8 mg/d for calcium and 1127±38.8 mg/d for phosphorus [16] but fall below the DRI of 1300 mg/d for calcium and 1250 mg/d for phospohorus [15].

**Table 2:** Dietary intake including micronutrients that were significantly different between 6 mo and 1 wk.

	DRI	Av US intake	6 mo			1 wk			P-value
			Actual Daily Intake	%DRI	% Av US intake	Actual Daily Intake	% DRI	% Av US intake	
Energy (kCal)		1861	1403.7±152.9		75.4	1271.6±231.2		68.3	0.4798
Protein (g)	46	65.6	59.9±6.1	130.2	91.3	49.6±8.0	107.8	75.6	0.2283
*Fat (g)	*35	69.2	41.2±5.3	117.7	59.5	46.1±13.3	131.7	66.6	0.6634
Carbohydrate (g)	130	248	204.5±26.0	157.3	82.5	173.5±29.4	133.5	70.0	0.1407
Linoleic Acid (g)	11	12.7	8±0.9	66.7	63.2	1.4±0.5	11.7	11.1	<0.0001
Copper (mg)	0.69	1	2.2±0.2	321.2	220.0	1±0.1	146.0	100.0	0.0117
Zinc (mg)	7.3	9.6	16.8±1.4	230.1	175.0	7.4±1.1	101.4	77.1	0.0031
Iron (mg)	7.9	13.8	22.6±2.1	286.1	163.8	11.4±1.5	144.3	82.6	0.0156
Niacin (mg)	11	20.8	27.5±2.3	196.4	132.2	15.2±2.0	108.6	73.1	0.0086
Pantothenic Acid (mg)	5		9.3±1.0	186		4±0.8	80.0		0.007
Riboflavin (mg)	0.9	1.78	2.6±0.2	260	146.1	1.4±0.2	140.0	78.7	0.007
Vitamin B12 (µg)	2	4.14	7.4±0.6	370	178.7	3.1±0.5	155.0	74.9	0.0144
Retinol (µg)	700	422	3642±405.5	520.3	863.0	346.3±77.9	49.5	82.1	<0.0001
Vitamin A (IU)	1616	1760	12975.5±1447.8	802.9	737.2	751.2±125.4	46.5	42.7	<0.0001
Vitamin D (IU)	400	152	353.7±35.6	88.4	232.7	3.6±0.8	0.9	2.4	<0.0001

P-value describes the difference in actual intake at 1 week and 6 mo. All micronutrient p-values have been adjusted for multiple comparisons using the Bonferroni method.

DRI, dietary recommended intake for females 14–18 years from United States Department of Agriculture (UDSA) based on National Academy of Sciences, Institute of Medicine, Food and Nutrition Board recommendations. (http://fnic.nal.usda.gov/nal_display/index.php?info_center=4&tax_level=3&tax_subject=256&topic_id=1342&level3_id=5140).

Av US intake, average dietary intake for females 12–19 years from USDA NHANES survey data 2007–2008 (http://www.ars.usda.gov/Services/docs.htm?docid=18349).

* AMDR, Acceptable Macronutrient Distribution Range, which is the range of intake for an energy source that is associated with reduced risk of chronic disease while providing adequate intakes of essential nutrients.

## Discussion

Subject report of significantly increased physical activity, with no change in energy intake, preceded inpatient medical hospitalization for female adolescents with AN. In addition, no change in the macronutrient content of the diet was reported over the six month period preceding hospitalization for subjects in this study. This rejects the hypothesis that there would be progressive energy restriction involving selective depletion of fat and carbohydrate intake prior to inpatient medical hospitalization for AN. However, the hypothesis that inpatient admission would be preceded by an increase in physical activity was confirmed. It must be noted that this study measured only the biological contributions of dietary intake and physical activity, without examining the psychological factors that play a role in the refusal of patients to take steps to ameliorate the catabolic state of AN. Undoubtedly, these psychological factors are important determinants of the need for inpatient medical treatment but were not examined in the present study.

Data showing no change in overall energy or macronutrient intake over six months may indicate that maximal dietary restriction happened more than six months prior to medical admission. Thus, these AN patients have lower energy intake than age matched counterparts (Table 2) but their intake did not decrease over the time frame of this study. The median duration of illness in this cohort was 6 months, indicating that abnormal eating behavior and weight loss had been observed prior to the period of time evaluated in this study. A previous study has shown that decreases in total caloric intake can be detected up to one year prior to the onset of AN compared to control subjects with the only difference in energy intake in AN patients over time appearing between one year and diagnosis [10]. Therefore, this study was not of sufficient duration to describe changes in dietary intake that are associated with the onset of AN. A significant limitation of this study is that the data analyzed was based on subject recall and reporting at the time of medical hospitalization. Data about weight or rate of weight loss during the corresponding time frame was not part of the study, and the retrospective nature of the dietary recall does not provide data that might be more accurate if collected prospectively in the course of outpatient monitoring of AN. Future studies should attempt to gather this information.

Our estimate of 1271.6±231.2 kcal/d for energy intake at one week closely matches that from previous studies in patients acutely presenting for treatment [11], [13]. Our 6 mo data for intake (1403.7±152.9 kcal/d) also closely mimics that from a prospective study which showed that AN girls had an intake of 1446±417 kcal/d at initial diagnosis [10]. These data are significantly lower than the intake reported for AN adolescents who are free living and do not need inpatient medical treatment (1649±110 kcal/d [14]) or for recovered AN patients (1942±543 kcal/d [8]), as would be expected. The macronutrient intake described herein is similar to previous reports [8], [10], [11] and not different from the US average intake [16].

The finding that acute changes in physical activity, not diet, contributed to the severity of malnutrition which precipitated inpatient medical admission for AN is corroborated by other retrospective studies that used categorical rather than quantitative measurements to show that 50–80% of AN patients report excessive physical activity prior to admission for treatment [21], [22], that there is a significant correlation between physical activity and the choice of “healthier” foods [23], and that physical activity has direct effects on both body weight and dieting practices [24]. As early as 1978, it was suggested that hyperactivity was a key feature of AN which may be the most resistant to resolve during treatment [21]. Data shows that 57% of AN patients reported that increased physical activity occurred before changes in dietary intake [22]. This high amount of self-reported physical activity is substantiated by studies using doublely labeled water to measure total energy expenditure which found that AN patients are engaged in about four times as much sports activity as controls [25] and that total energy expenditure from physical activity for AN patients is double that for controls [26]. Only one study conducted to date, using accelerometers to measure physical activity in a broad range of AN patients, from actively receiving refeeding therapy to recovered, showed no difference in physical activity between AN patients and controls [27]. This result may have been due to the fact than many recovered AN subjects were included who have lower total activity. Therefore, it is important for clinicians to focus on this aspect of behavior once an AN diagnosis is made. Careful monitoring and efforts to appropriately limit physical activity following AN diagnosis may reduce the need for inpatient medical care.

Only one previous study quantitated the amount of physical activity in free-living AN subjects and reported 15.7±1.6 MET/d [14] which is considerably lower than that observed in this study at one week but is directly comparable to the level that we documented over 6 months prior to admission when our subjects were free living and most likely had a greater resemblance to those in a community setting. The discrepancy in physical activity estimates at one week could also be due to the fact that the previous study [14] was conducted with AN subjects from the community under general outpatient medical care who may have had less severe AN symptoms than those in our study who required inpatient medical care.

The strong association between reported physical activity and inpatient medical admission for AN further validates the activity-based AN model in rats [28], [29], [30]. In rats, restriction of food intake by time but not total amount offered causes a growth plateau but not spontaneous AN. However, under this same limited time with food paradigm, the addition of a running wheel for voluntary physical activity causes decreased food intake and progressive increases in physical activity, a phenomenon referred to activity-based AN [29]. This model is analogous to our study results, as no change in diet but increased physical activity leads to overt AN in both humans and rats. Therefore, it would be valuable to use the data presented herein and apply them to the existing activity-based AN model to more closely mimic the human condition. This model would then be a valuable screening tool for dietary manipulations that may be more effective for the refeeding treatment of AN.

In this study, adolescent girls with AN reported deficient intake of Vitamin D and linoleic acid over 6 months; and deficient intake of Vitamin A, Vitamin D, linoleic acid, retinol, and pantothenic acid at 1 wk prior to admission. This is in contrast to previous data showing that adult AN subjects had higher intakes of Vitamins A and D than healthy, control subjects due to the use of supplements [14]. Our data reflects overall intake from food plus supplements and still shows significant Vitamin A and D deficiency which implies a lower use of vitamin and mineral supplements in our adolescent population. Indeed, in our data supplement usage was very heavy over 6 mo (90% of subjects reported taking supplements; overall average of 6.6±0.9 pills/week) whereas use had dwindled dramatically at one week prior to admission (one subject, 5%, of cohort took 2 pills/week; overall average of 0.1 pills/week). A limitation of this study was not having serum measurement of micronutrients at the time of admission. This was beyond the scope of the funding for this study, but would be useful to include in future studies.

Our quantitative data for micronutrient intake matches very closely with that described previously [14]. Deficient Vitamin D intake was prevalent over 6 months prior to medical admission, at which time it was mild according to DRI and actually above the average US intake for females aged 14–18 years [15] (Table 2). However, a gross decrease in Vitamin D intake occurs between six months and one week prior to admission causing severely deficient intake (0.9% of DRI, 2.4% of average US intake) at one week. In a previous study, it was reported that only 26% of AN subjects met the DRI for Vitamin D intake [14]. Rickets, or insufficient bone mineralization during growth, is most commonly associated with deficient Vitamin D intake but other effects include decreased nervous system function and lower bone density due to low calcium absorption [31]. These effects are of specific concern in AN patients so Vitamin D intake should be assessed and monitored during AN treatment as a surrogate marker of disease severity.

Results from dietary intake for 6 months prior to admission showed Vitamin A intake exceeded the DRI by more than 800% but dropped dramatically to only 47% of DRI [15] (43% of average US intake) at 1 week, which is low enough to cause decreased growth rate, slower bone development, and decreased immunity [32]. The upper limit of vitamin A intake for females aged 14–18 years is 9,335IU/d but the population examined in this study had an intake of 12,975IU/d. This high intake of vitamin A is in excess of the quantity needed to cause hypervitaminosis A which is associated with nausea, loss of appetite, dizziness, cerebral edema, and, in older adults, osteoporotic fracture [32]. These symptoms (nausea, dizziness, reduced appetite) and decreased bone density are of importance in the evaluation and treatment of AN. At the time of inpatient medical admission, Vitamin A intake had decreased dramatically to 47% of DRI. Thus, it seems necessary to monitor Vitamin A status from the time of AN diagnosis through weight restoration in order to establish safe levels in AN patients.

Several other micronutrients were reported to have elevated intake over the six months prior to admission including zinc, copper, iron, and most B group vitamins but these intakes did not exceed the recommended upper limit. For example, at an intake of 22.6±2.1 mg/d, iron intake over six months is 286% of the DRI. However, this level does not reach the recommended upper limit of intake which is 45 mg/d for females aged 14–18 years. Folate and pantothenic acid intakes were reported within normal ranges over six months prior to hospitalization, but fall below the DRI at one week prior to hospitalization. Folate deficiency causes megaloblastic anemia whereas pantothenic acid deficiency is rare in humans.

A significant limitation of this data is that this was a small study which relied on self-report to measure dietary intake and physical activity levels. Due to the nature of the instruments used to measure dietary intake and physical activity in this study, it is not possible to measure progressive changes over time as these questionnaires can only collect data cumulatively over 6 months and then acutely one week prior to admission. Additionally, the questionnaires administered cannot determine the behavioral aspects of dietary intake such as meal patterns, the proportion of food consumed during the night, or the timing of food intake relative to exercise bouts. These are important factors which should be considered in future studies. Also, self-reported dietary intake is notoriously inaccurate in healthy and obese individuals who tend to under-report dietary intake [33]. The major error associated with self-report in these populations stems from inaccurate estimation of portion sizes [34]. However, patients with AN are acutely aware of portion sizes and nutritional information for every food which they ingest. Therefore, it is reasonable to assume that the bias in self-reporting would be towards *over-reporting* rather than the under-reporting associated with other well studied populations. There is little data directly supporting this assumption as the three studies conducted thus far have reported conflicting results [35]–[37].

However, 24 h recall data argues against the idea of under-reporting in this population. Energy intake calculated from the 24 h recall (1,272±237 kcal) is comparable to data reported from studies conducted under similar conditions in adolescents (1,150±110 kcal [13]) and young women (mean 23.2 year, 1,017±54 kcal [11]) and very closely matches the calculated daily caloric needs for weight stability in our subjects (1257.2±23.6 kcal; Table 1). Given that physical activity increased significantly acutely prior to inpatient medical admission for AN, it is feasible to assume that dietary intake was sufficient to meet minimal daily caloric needs and the increased rate of weight loss was caused by the marked increase in physical activity. Therefore, this data supports both the hypothesis that increased physical activity leads to inpatient medical admission for AN, and that self-reported dietary intake is relatively accurate in this population. Validation studies using doubly labeled water are required to corroborate this assumption [33].

### Conclusions

Increased physical activity, without any change in energy or macronutrient intake, was associated with inpatient medical admission for the treatment of AN in adolescent females. Although there was no reported change in energy or macronutrient intake over the 6 month period of this study, micronutrient intake changed which could lead to inadequate intake of Vitamin D acid over 6 months; and deficient intake of Vitamin A, Vitamin D, retinol, and pantothenic acid at 1 wk. Over 6 months prior to admission, reported Vitamin A intake was at extremely high, potentially harmful, levels. These results suggest that, in addition to the careful monitoring of weight and overall energy intake, evaluation, monitoring and treatment interventions for AN should include monitoring physical activity, Vitamin A, and Vitamin D status as markers of disease severity and progression.
